# Anogenital Lesions: Kaposi's Sarcoma and Its Mimicks

**DOI:** 10.5402/2012/486425

**Published:** 2012-10-31

**Authors:** Louis-Jacques van Bogaert

**Affiliations:** National Health Laboratory Service, Polokwane/Mankweng Hospital Complex and University of Limpopo, Groblersdal 0470, South Africa

## Abstract

Kaposi's sarcoma (KS) is a low-grade vascular neoplasm associated with human herpes virus-8 (HHV-8) infection, and, in the epidemic form, with the human immunodeficiency virus (HIV). Although HHV-8 is present in all body fluids and is sexually transmitted, there are surprisingly few reports of anogenital KS. Clinically, especially in HIV/KS endemic areas, dark stained skin patches or nodules are prone to misdiagnosis, especially in dark-skinned individuals. Therefore, a biopsy is recommended. The histologic appearance spans a broad spectrum of KS and non-KS lesions; therefore, the final diagnosis should be confirmed by HHV-8 immunohistochemistry. We report a series of 36 anogenital biopsies from a group of 16 documented HIV-positive patients; in 20 the HIV serostatus was unknown. There were ten KS (five in HIV-positive patients), and 26 non-KS (11 in HIV-positive subjects) lesions. In the era of HIV/AIDS, anogenital lesions may be the first manifestation of KS in immunocompromised individuals and should be biopsied. The histological diagnosis should be confirmed by HHV-8 immunohistochemistry.

## 1. Introduction

The human herpes virus-8 (HHV-8), also called Kaposi's sarcoma herpes virus (KSHV), is the causative agent of all types of Kaposi's sarcoma (KS), namely the classic, endemic, iatrogenic immunosuppression, and the epidemic/human immunodeficiency virus (HIV) related forms. The modes of acquisition of the KSHV and its transmission vary with age, gender, geography, and sexual practices [1]. Although the virus is omnipresent its incidence is highest in KS endemic regions such as sub-Saharan Africa (sSA).

In Uganda, HHV-8 DNA was detected in 28 percent of oral swabs and 27 percent of blood samples of healthy asymptomatic subjects [2]. Hence, saliva is a potential source of transmission. KSHV is transmitted to children from maternal and nonmaternal sources in KS-endemic regions, and occurs via nonsexual routes [3–6]. It has been shown that, in sSA, infection occurs during childhood mainly via maternal saliva and breast milk [7, 8]. Anthropologic research in sSA has identified that premastication of foods of infants and children is traditional, and that saliva, semen, and vaginal fluids are used while engaging in nonsexual practices associated with childcare [9–11].

The sexual transmission of KSHV is more controversial. In the industrialized world, there is evidence of transmission between men having sex with men (MSM) via anal but not oroanal sex [7, 12, 13]. Heterosexual transmission is affirmed by some [14–18]. Others deny it [5, 19]. Since KSHV is present in semen and in uterine cervical scrapings, sexual transmission appears to be very plausible [17, 20, 21]. However, despite the high incidence of KSHV infection and its presence in the male and female genital tract, surprisingly few cases of anogenital KS have been reported in the literature, associated or not with HIV coinfection, in males or females [22–27].

The purpose of this study was to describe the clinical and histopathological spectrum of anogenital lesions either under- or overdiagnosed as KS in a HIV/AIDS endemic province of South Africa.

## 2. Materials and Methods

The study was carried out at the Histopathology Department of the National Health Laboratory Service of the Limpopo Province. The population is mainly rural and is in excess of 5 M. The department receives and reports all the histology specimens from the public hospitals in the province.

**Figure 1:**
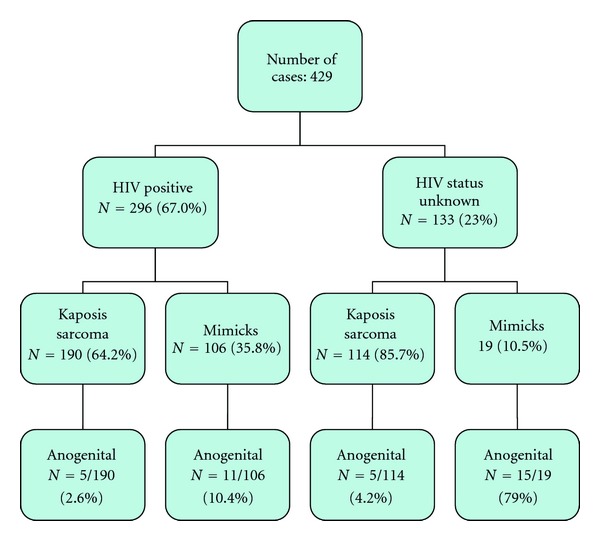
Flow chart of the distribution of cases.

The cases were retrieved from a prospective survey of 429 biopsy and LAN-1 immunohistochemically stained cases from March 2010 through July 2012 (Figure 1). Patients' data were recorded from the biopsy request form and the laboratory data base. The following information was compiled: age, gender, clinical information, clinical provisional diagnosis, and HIV serostatus.

Routine streptavidin-biotin-peroxidase immunostaining with diaminobenzidine was performed on formalin-fixed paraffin-embedded tissue using a murine monoclonal antibody directed against the C-terminus of the latent nuclear antigen-1 (LNA-1) molecule of HHV-8 (clone 13B10; Novocastra, Newcastle upon Tyne, UK).

Patients' anonymity was preserved. Ethical approval was obtained from the institutional research ethics committee.

## 3. Results

Figure 1 illustrates the distribution of cases by HIV status, immunohistochemically diagnosed KS, and mimicks. We collected a total of 36 cases of anogenital lesions: 15 in males and 21 in females. Sixteen (44.4%) had a documented HIV infection; 20 were of unknown HIV serostatus.

Table 1 shows the clinicopathology of male anogenital lesions. There were 4 KS and 11 mimicks; four (26.6%) were HIV-positive. Pyogenic granulomas accounted for 7 (46.6%) of non-KS lesions. KS was clinically suspected in one case only.

Table 2 illustrates the clinicopathological presentation of female anogenital lesions. Twelve (46.2%) were known to be HIV-positive. The clinical diagnosis of KS was made in cases with known disseminated KS.

## 4. Discussion

African endemic KS has been identified for many decades in the past. Currently, the increasing prevalence of HIV infection complicates all efforts to clearly distinguish the endemic variant from the HIV-related one [28]. The clinical presentation and natural history of the endemic KS are now blending with those of the epidemic or AIDS-associated disease [29]. The distinction is clinically relevant because, although it is amenable to highly active antiretroviral treatment (HAART), AIDS-associated KS carries a poor prognosis [30]. Unfortunately, in South Africa, there is still widespread reluctance to be tested for HIV. This is attributable to the official opt-in policy (voluntary counseling and testing), and the fear of stigma and discrimination. This adds to the difficulty in distinguishing endemic from epidemic KS cases.

Early reports on African KS concentrated on the gender differences and histological features [31, 32]. One publication indicated that the most common presentation was cutaneous; it emphasised the rarity of anogenital location [33]. In a textbook of 1957, Bluefarb cited six patients with KS of the glans penis [34]. A series of 29 KS cases retrospectively collected from 1973 till 1985 in Uganda mentioned external genital involvement of 15 [35]. A Nigerian report mentioned that anogenital involvement was more common in HIV-positive cases [36]. A more recent publication of 66 biopsy-confirmed KS and HIV-seropositive patients found 10 (15.2%) genital sites (not otherwise specified); no genital KS was found in 11 cases of endemic KS [28]. Among twenty biopsy-proven Nigerian KS, the penis and rectum were involved in what appears to be cases of disseminated KS [37]. In an Ugandan series of 197 HIV-associated KS (only 62% biopsy-proven) it was reported that 6% were located on the genitals (no gender distribution or precise anatomic location); half of the series had lesions in two or more anatomic locations [38]. The difficulty with most of these reports is that the anogenital location seems to have been part of multicentric or disseminated KS, and that none was LNA-1 confirmed. 

It is now well established that both the clinical and histological diagnosis of KS is fraught with over- and underdiagnosis because of the wide range of mimickers [39, 40]. Therefore, especially in HIV/AIDS and KS endemic areas, a clinical lesion suspect of KS should be biopsied, and the histopathological diagnosis must be supported by HHV-8 immunohistochemistry. Clinically, the most common mimicks are seborrheic keratosis, haemangioma, and pyogenic granulomas. Histopathologically, around thirty lesions are part of the differential diagnosis [39, 40]. Seborrheic keratosis mimicks early patch stage KS. Pyogenic granulomas and haemangioma mimick nodular stage KS. Fibrous histiocytoma and tendosynovitis mimick the plaque stage, to name only a few and most common ones.

Literature case reports all illustrate that vulvar KS appeared clinically as a mass, a papilloma, or an abscess; none was initially suspected to be a KS [22–26]. In the present series, KS was clinically diagnosed only in one instance; one was overdiagnosed microscopically as KS before LAN-1 immunostaining.

In conclusion, anogenital KS is rare and easily misdiagnosed even in HIV/KS endemic regions. Therefore, a high clinical suspicion threshold should prompt a biopsy, and the diagnosis of KS should be confirmed by LNA-1 immunohistochemistry to avoid misdiagnosis and wrong management.

## Figures and Tables

**Table 1:** Clinicopathological presentation of anogenital lesions in males.

Age	HIV Status	Clinical Presentation	Histopathology
54	Unknown	Scrotal mass	Nodular stage KS
38	Positive	Perineal ulcer	Plaque stage KS
29	Unknown	Penile ulcer	Nodular stage KS
35	Unknown	Penile KS	Early patch stage KS
49	Unknown	Scrotal ulcer	Pyogenic granuloma
34	Positive	Scrotal ulcer	Pyogenic granuloma
27	Unknown	Scrotal pyogenic granuloma	Pyogenic granuloma
72	Unknown	Scrotal ulcer	Pyogenic granuloma
30	Unknown	Penile ulcer	Pyogenic granuloma
59	Positive	Perianal ulcer	Pyogenic granuloma
63	Unknown	Perianal ulcer	Pyogenic granuloma
47	Unknown	Perianal nodule	Hemangioma
24	Unknown	Scrotal nodule	Hemangioma
32	Positive	Fournier's gangrene	Fournier's gangrene
41	Unknown	Penile nodule	Malignant fibrous histiocytoma

**Table 2:** Clinicopathologic presentation of anogenital lesions in females.

Age	HIV Status	Clinical Presentation	Histopathology
48	Positive	Vulvar mass	Nodular stage KS
31	Positive	Vulvar mass (disseminated **KS**)	Nodular stage KS
33	Unknown	Cervical erosion	Nodular stage KS
34	Positive	Vulvar nodule	Early patch stage KS
24	Unknown	Vulvar nodule	Nodular stage KS
24	Positive	Vaginal **KS**	Nodular stage KS
19	Positive	Vulvar **KS**	Pyogenic granuloma
48	Unknown	Anal ulcer	Pyogenic granuloma
40	Unknown	Vaginal ulcer	Pyogenic granuloma
35	Positive	Vulvar nodule	Pyogenic granuloma
44	Positive	Vulvar nodule	Pyogenic granuloma
22	Negative	Vulvar nodule	Pyogenic granuloma
44	Positive	Anal ulcer	Pyogenic granuloma
25	Positive	Vulvar nodule	Fibrous histiocytoma
25	Positive	Vulvar carcinoma	Fibrous histiocytoma
49	Unknown	Vulvar nodule	Fibrous histiocytoma
58	Unknown	Cervical mass	Pedunculated leiomyoma
49	Unknown	Vulvar violaceous lesion	Lichen planus
42	Positive	Vulvar dark skin patch	Melanocytic melanoma
45	Positive	Vulvar nodule	Hemangioma
43	Unknown	Cervical erosion	Cervical cancer
